# Tracción de un incisivo central superior en posición ectópica con un aparato de hawley modificado: reporte de un caso clínico

**DOI:** 10.21142/2523-2754-1001-2022-101

**Published:** 2022-03-30

**Authors:** Tony Sánchez-Achío

**Affiliations:** 1 Departamento de Odontopediatría y Ortodoncia, Universidad de Costa Rica. San José, Costa Rica Universidad de Costa Rica Departamento de Odontopediatría y Ortodoncia Universidad de Costa Rica San José Costa Rica; Universidad Intercontinental. Ciudad de México, México. dr_tsanchez@hotmail.com Universidad Intercontinental Ciudad de México México dr_tsanchez@hotmail.com

**Keywords:** ortodoncia, tracción, incisivo, odontoma, orthodontic, traction, incisor, odontoma

## Abstract

La prevalencia de la impactación de los incisivos maxilares permanentes oscila entre un 0,06 y un 1% de la población. Una de las posibles causas de impactación del incisivo central superior es la presencia de un odontoma. El estudio de este caso clínico tuvo como objetivo plantear una propuesta de tratamiento ortodóncico para una niña con dentición mixta temprana, mediante la tracción de un incisivo central maxilar impactado, problema poco común en la población, causado por la presencia de un odontoma compuesto. Paciente femenina de 6 años y 2 meses de edad se presentó a la consulta privada. El examen clínico mostró un retraso en la exfoliación de las piezas dentales 5.1 y 5.2; la radiografía panorámica reveló la presencia de un odontoma compuesto y la tomografía mostró la pieza dental 1.1 en una posición ectópica, horizontal en el plano sagital. Se realizó la remoción del odontoma compuesto al mismo tiempo que se expuso el diente impactado, y se decidió utilizar un aparato removible tipo Hawley modificado con una torre para realizar la tracción del diente. La elección de buenos registros diagnósticos es esencial en el diagnóstico temprano y oportuno de patologías óseas y alteraciones dentales. La paciente presentó una impactación del diente 1.1 y, debido a su dentición mixta temprana, no fue posible tratarla convencionalmente con aparatología fija de *brackets*, por lo que se decidió realizar un aparato removible tipo Hawley modificado. El tiempo de tratamiento de la tracción dental fue de 8 meses, tiempo relativamente corto, según la literatura.

## Abstract (en)

The prevalence of impaction of the permanent maxillary incisors ranges from 0.06 to 1% of the population. One of the possible causes of impaction of the upper central incisor is the presence of an odontoma. The objective of this clinical case study was to propose an orthodontic treatment proposal for a girl with early mixed dentition, pulling an impacted maxillary central incisor, a rare problem in the population, caused by the presence of a compound odontoma. A 6- year-old and 2-month-old female patient presented to the private consultation. On clinical examination, she showed a delay in exfoliation of the teeth 5.1 and 5.2, the panoramic radiograph revealed the presence of a compound odontoma. And in the tomography, the dental piece 1.1 was observed in an ectopic position, horizontal in the sagittal plane. The composite odontoma was removed at the same time as the retained tooth was exposed, and it was decided to use a modified Hawley, type removable appliance with a tower to perform traction of the tooth. The choice of good diagnosis of bone pathologies and dental alterations. The patient presented an impaction of tooth 1.1 and due to her early mixed dentition, it was not possible to treat it conventionally with fixed bracket appliances, reason why it was decided to make a modified Hawley type removable appliance. The treatment time for dental traction was 8 months, a relatively short time, according to the literature.

## INTRODUCCIÓN

Un diente se considera impactado cuando no ha erupcionado en el tiempo que se espera y no logra salir espontáneamente por diferentes motivos obstructivos. Radiográficamente, se podría identificar porque dos tercios de su raíz están desarrollados, pero algo le impide erupcionar naturalmente ^1^. El grupo de dientes que presenta más impactación son los terceros molares con un 98%, caninos superiores con una frecuencia del 1,3%, primeras premolares inferiores 0,22% y segundos premolares inferiores 0,11% ^2^^,^^3^.

La prevalencia de la impactación de los incisivos maxilares permanentes se presenta entre el 0,06% y el 1% de la población, según el autor ^4^^-^^6^. Las causas de este fenómeno pueden ser traumáticas, ocasionando una alteración en el brote dental; obstructivas, usualmente por un diente supernumerario o un odontoma; también se pueden presentar quistes, otros tumores, dientes primarios con retraso en su exfoliación o mucoperiostio denso; y otras, que pueden ser la dilaceración de la raíz del diente, síndromes, anquilosis dental o una morfología anormal del incisivo ^7^^-^^8^. 

La mayoría de los incisivos impactados presentan complicaciones clínicas como posición ectópica del diente en el 46,6%, pérdida de espacio en el 36,9% y desplazamiento de la línea media en el 27,5% de los pacientes ^9^.

En caso de sospechar la impactación de un incisivo maxilar, es importante verificar la erupción tardía de los incisivos maxilares, prestar atención si el diente contralateral erupcionó hace más de 6 meses, si ambos incisivos centrales maxilares permanecen sin erupcionar y los incisivos inferiores lo han hecho hace más de un año, o si existe otra alteración en la secuencia de erupción dental. Clínicamente, cuando no se presenta el incisivo central maxilar permanente en boca, es usual pensar en la impactación de esta pieza dental, ya que su falta congénita es sumamente rara, con una prevalencia del 0,01% ^6^. 

Las radiografías convencionales, como las panorámicas, se recomiendan como protocolo ya que mostrará toda la dentición del paciente y, con respecto a la retención de incisivos permanentes maxilares, pueden revelar la existencia de un diente impactado, el grado de resorción de la raíz del diente primario y la profundidad de la impactación, para brindar un diagnóstico certero. Sin embargo, se puede generar confusión si se superponen imágenes como el paladar duro y el hueso cigomático ^6^^,^^7^. La tomografía axial computarizada es el método de elección para el diagnóstico completo de un diente impactado, porque brinda más información que la radiografía panorámica, como la ubicación exacta del diente; además, se pueden observar y medir problemas en la zona como patologías óseas o reabsorciones de dientes adyacentes, ofreciendo una clara ventaja a la hora de realizar el tratamiento quirúrgico y ortodóncico ^7^^-^^8^^,^^10^^-^^11^.

La evidencia científica enfatiza en la importancia del diagnóstico precoz de un incisivo maxilar permanente impactado y la intervención en el momento adecuado. Entre más temprano se establezca el diagnóstico, aumenta la posibilidad de lograr mejores resultados y evitar complicaciones, como la pérdida del potencial de erupción, la desviación de línea media, la pérdida de espacio, los cierres apicales prematuros, los problemas psicosociales, las dilaceraciones de las raíces de las piezas dentarias cercanas, los problemas periodontales, la formación de quistes y los tratamientos ortodóncicos más extensos ^2^^,^^5^.

Entre las opciones de tratamiento para los incisivos centrales maxilares impactados está la exposición quirúrgica con extrusión ortodóncica. La tracción ortodóncica se puede realizar con aparatología fija o removible para posicionar un incisivo central maxilar impactado en el lugar indicado, lo que representa un gran desafío, debido a su posición estética que también involucra los tejidos duros y blandos adyacentes ^7^^-^^9^^,^^10^^-^^17^. 

El pronóstico del tratamiento quirúrgico y tracción ortodóncica para incisivos centrales impactados es bueno; sin embargo, es largo en el tiempo, hasta 2 años, según Chaushu *et al*. ^7^. Además, se ve significativamente afectado por la altura inicial del diente impactado, la posición y la dirección del diente, la edad del paciente, la dilaceración, entre otras ^8^^,^^10^. 

Una de las posibles causas de la impactación de un incisivo central superior es la presencia de un odontoma, que con frecuencia se asocia a dientes deciduos con retraso de exfoliación, los cuales interfieren con la erupción de los dientes permanentes ^16^^,^^17^. El odontoma es el tumor odontogénico benigno más común y representa del 22% al 40% de todas estas lesiones; son malformaciones tumorales epiteliales y mesenquimales mixtas compuestas de todos los tejidos duros y blandos de un germen dental maduro (esmalte, dentina, cemento y tejido pulpar) ^18^. No tienen predilección por ningún sexo y la etiología es desconocida, pero se relaciona con traumas, herencia, infecciones locales y mutaciones genéticas. Generalmente, son asintomáticos y se detectan en radiografías de rutina frecuentemente asociadas a un diente permanente no erupcionado ^12^^,^^13^^,^^16^^,^^19^.

Los odontomas se clasifican clínicamente en compuestos y complejos. Los odontomas compuestos son más comunes en la región anterior de los maxilares y aparecen como numerosos dientes de tamaño pequeño, llamados dentículos. Su crecimiento es lento y, radiográficamente, se observan como múltiples dentículos rodeados por un halo radiolúcido ^13^^,^^20^. Los odontomas compuestos son dos veces más frecuentes que los complejos. La remoción quirúrgica de ambos tipos es recomendada con la finalidad de evitar complicaciones locales ^13^^,^^18^^,^^19^.

El estudio de este caso clínico tuvo como objetivo plantear una propuesta de tratamiento ortodóncico para una niña con dentición mixta temprana, realizando la tracción de un incisivo central maxilar impactado, problema poco común en la población, causado por la presencia de un odontoma compuesto.

## REPORTE DEL CASO CLÍNICO

El presente caso clínico contó con un consentimiento informado, firmado por la madre de la paciente para que los datos de la historia clínica, imágenes, radiografías, aparatos ortodóncicos y cualquier tipo de información acerca del tratamiento de su hija pueda ser publicado en una revista científica o expuesto en un congreso odontológico, con fines científicos y docentes. 

**Figura 1:**
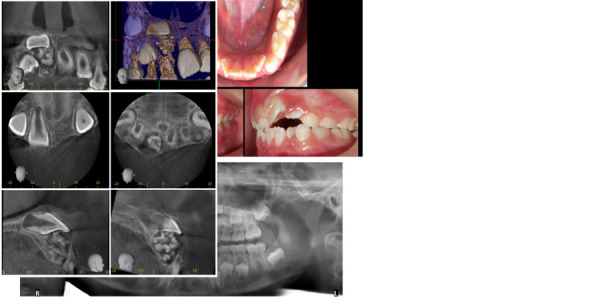
Registros diagnósticos iniciales de la paciente en estudio. A) Fotografías intraorales. B) Radiografía panorámica inicial. C) Imágenes tomográficas del sector anterosuperior

Paciente femenina de 6 años y 2 meses, de nacionalidad salvadoreña, se presentó a la consulta privada de ortodoncia en San José, Costa Rica, con la queja principal de su madre: “El diente del frente le está saliendo torcido”, refiriéndose al diente 2.1. Al examen clínico, se observó una maloclusión clase II, relaciones caninas temporales de clase I bilateral, sobremordida horizontal de 1 mm en la pieza dental 5.1 y una sobremordida vertical de -3mm (mordida abierta), por falta de erupción de las piezas dentales 2.1 y 2.2. Se observó un apiñamiento moderado en el sector anteroinferior. Con referencia a la queja principal, se observó el diente 2.1 con angulación mesial (inclinación de la raíz hacia mesial y la corona anatómica hacia distal), además de rotación mesial de los dientes 2.1 y 2.2. En el cuadrante 1 se observó la presencia de los dientes deciduos 5.1 y 5.2 (Figura 1.A). En esa cita se decide enviar la toma de una radiografía panorámica para observar la secuencia de erupción de todas las piezas dentales con énfasis en los dientes anterosuperiores, la existencia de patologías óseas, presencia de anomalías dentales de tamaño, número y forma, entre otras cosas.

La Figura 1.B. muestra la radiografía panorámica que evidenció en la zona apical del diente deciduo incisivo central maxilar derecho (5.1) una lesión unilocular con múltiples radiopacidades internas y límites definidos por una zona radiolúcida, compatible con un odontoma compuesto. Además, se observó una malposición del diente incisivo central maxilar derecho permanente (1.1). Debido a esto, se decidió enviar la toma de una imagen tomográfica de la zona.

La imagen tomográfica mostró en la zona apical de 5.1 múltiples cuerpos radiopacos sin forma definida, distribuidos de forma desorganizada, y presencia de límites bien definidos de la lesión, confirmando el diagnóstico de un odontoma compuesto. Coronalmente a la lesión, se observó la pieza dental 1.1 en una posición ectópica, ubicado horizontalmente en el plano anteroposterior o sagital, paralelo al paladar (Figura 1.C).

Inicialmente, se refirió la paciente a la cirujana maxilofacial para que le realizara la remoción del odontoma compuesto. El día de la cirugía, debido a la extensión de la lesión, la especialista tuvo que realizar las exodoncias de las piezas dentales 5.1, 5.2 y 5.3. Se aprovechó esa misma cita para exponer el diente 1.1 ubicado en posición ectópica. En esa exposición, se le adhirió al diente un botón que tenía amarrado una ligadura metálica de acero inoxidable 0,010” y se cerró el colgajo dejando por fuera la ligadura. Se dejó cicatrizar y desinflamar la zona por un mes, sin aplicar fuerza ortodóncica ni colocar ningún aparato en la zona. Posteriormente, la tracción se realizó a campo cerrado. 

**Figura 2:**
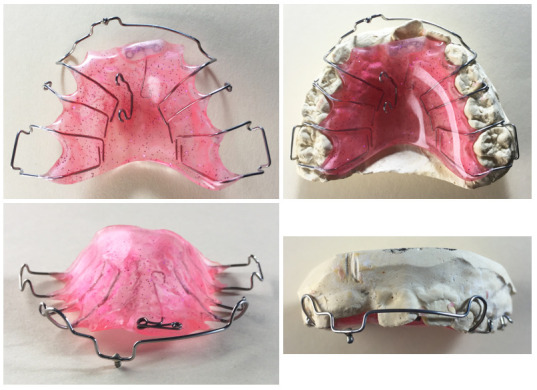
Aparato removible tipo Hawley modificado con una torre

Para la tracción del incisivo central maxilar derecho impactado (1.1), se decidió utilizar un Hawley modificado con una torre. Se trata de un aparato removible que está compuesto por una placa acrílica que cubre el paladar, posee aditamentos de retención (en este caso, ganchos tipo bola entre las molares temporales y Adams en molares permanentes) y un arco vestibular. A este aparato se le agregó una torre, que es un *step down* en el sector anterior del arco vestibular del Hawley con un botón hacia incisal, y dos ganchos de tracción en el paladar. Adicionalmente, se le colocó una doble acción para el diente 2.1, lo cual mejorará la mesiorrotación de esta pieza dental (Figura 2).

**Figura 3:**
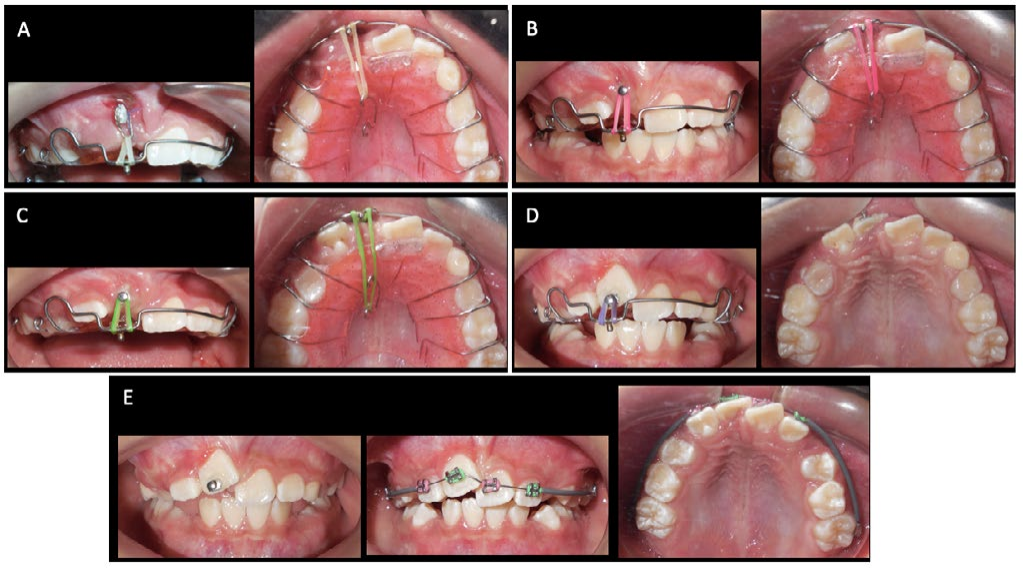
Tracción del incisivo central superior derecho permanente. A) Inicio de la tracción. B) 4 meses de tratamiento. C) 5 meses de tratamiento. D) 7 meses de tratamiento. E) 8 meses de tratamiento

Se instruyó a la paciente y a su madre para el uso y cuidado adecuado del aparato. Las indicaciones de uso fueron todos los días, todo el día, excepto para comer y lavarse los dientes, cuando tenía que removerlo y guardarlo en una caja destinada para este fin, utilizándolo aproximadamente 22 horas al día y colocando adicionalmente una liga de 3/16” con una fuerza de 4 onzas (onz) desde la ligadura hasta el gancho en el paladar, pasando por la torre (Figura 3.A). 

Cuatro meses después, se pudo observar la erupción del diente 1.2 y el botón pegado a la pieza 1.1. A partir de ese momento, se eliminó la ligadura y se siguió traccionando directamente del botón al gancho palatino. Progresivamente, se fue cambiando el anclaje de la tracción utilizando ambos ganchos en el paladar del Hawley y se utilizaron ligas 3/16” y 1/4” ambas de 4 y 6 onzas, para ir aumentando la fuerza de tracción (Figuras 3.B y 3.C).

A los siete meses de iniciado el tratamiento, se toma otra radiografía panorámica donde se observa una excelente verticalización del diente 1.1, y clínicamente ya se observa gran cantidad de su corona clínica (Figuras 3.D y 4.B). 

Ocho meses posteriores al inicio de la tracción, se suspendió el uso del Hawley y se colocó un aparato fijo 2x4, que consiste en 2 tubos adheridos a las primeras molares superiores permanentes y 4 *brackets* en los incisivos superiores permanentes, para mejorar la posición de los dientes anteriores, lo que dio por terminada la tracción del diente 1.1 (Figura 3.E).

**Figura 4:**
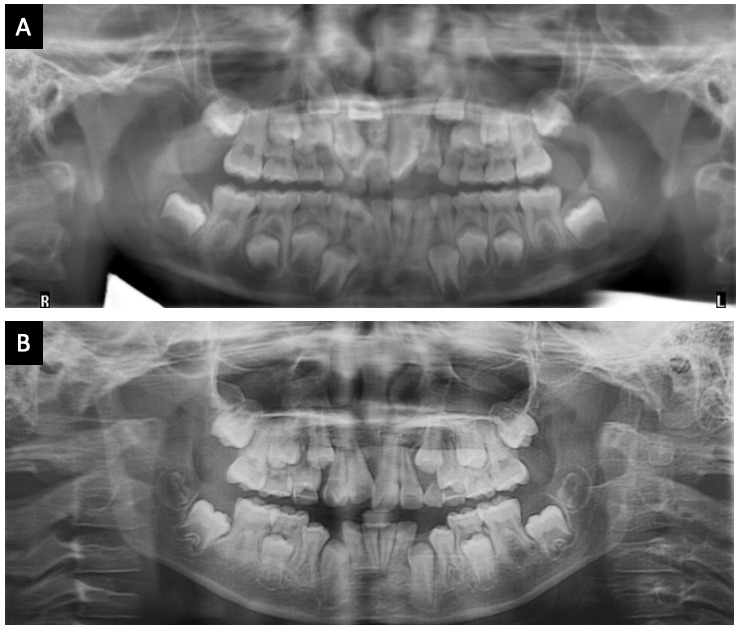
Radiografías panorámicas comparativas. A) Radiografía panorámica inicial. B) Radiografía panorámica a los 7 meses de tratamiento

La Figura 4 muestra los cambios de la radiografía panorámica inicial, con la presencia del odontoma compuesto, un retraso en la exfoliación de los dientes 5.1 y 5.2, además de la posición ectópica del incisivo central maxilar derecho permanente (1.1). A la radiografía con 7 meses de tratamiento, sin el odontoma compuesto y con el diente 1.1 en posición vertical. 

**Figura 5:**
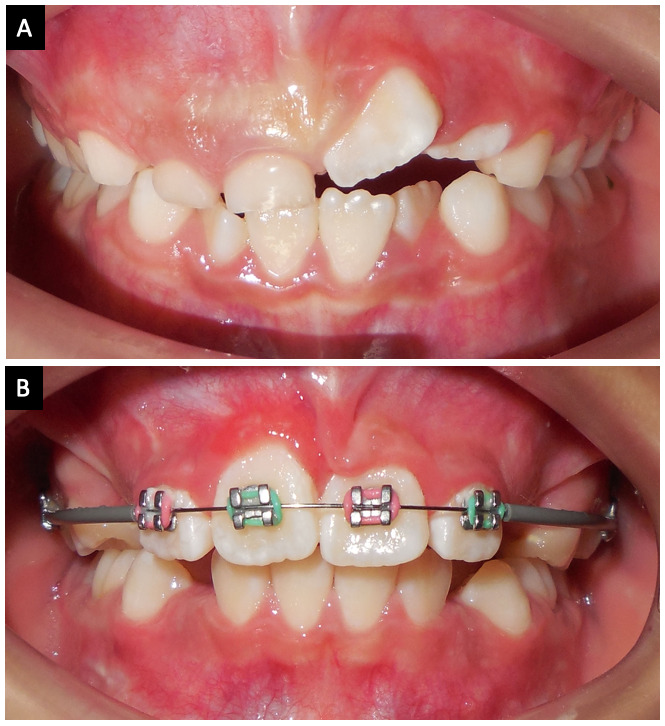
Fotografías intraorales comparativas. A) Fotografía intraoral inicial. B) Fotografía intraoral a los 9 meses de tratamiento

La Figura 5. A es una fotografía clínica antes del tratamiento, donde se evidencia la presencia y un retraso en la exfoliación de los dientes 5.1 y 5.2, además de la malposición de los dientes 2.1 y 2.2. La Figura 5.B es una vista clínica 9 meses después del tratamiento ortodóncico, con los cuatro incisivos maxilares permanentes erupcionados en proceso de alineación y un aparato ortodóncico de 2 x 4. Es importante aclarar que, del octavo al noveno mes, solo se realizó un alineamiento dental de los incisivos maxilares permanentes con un arco de NiTi redondo 0.014”. Por lo anterior, se considera que el proceso de tracción dental del 1.1 se efectuó en 8 meses.

## DISCUSIÓN

Paciente femenina de 6 años y 2 meses de edad se presentó con su madre a la consulta dental privada de ortodoncia. Mediante el examen clínico intraoral se pudo observar la presencia de los dientes 5.1, 5.2, 2.1 y 2.2 en el sector anterior superior, que presentaban una alteración en la secuencia de erupción ^6^, por lo que se decidió tomar registros radiográficos para realizar una completa valoración.

La importancia de un buen diagnóstico es esencial para realizar un adecuado tratamiento odontológico. En este caso, se concuerda con los autores anteriormente mencionados que, posteriormente a la radiografía panorámica, en presencia de patologías óseas y dientes impactados, se debe realizar un estudio tomográfico de la zona anterior del maxilar, con el fin de observar la localización y extensión de la patología presente, además de la ubicación del incisivo impactado ^7^^,^^8^^,^^10^.

La radiografía panorámica y la tomografía axial computarizada se utilizaron para el diagnóstico del odontoma compuesto ya que, según la literatura, son los registros diagnósticos idóneos para la detección de su presencia, puesto que la presencia de esta patología es asintomática ^12^^,^^13^^,^^16^^,^^19^. Se coincide y procede según los criterios de Niharika et al. ^13^ y Brenes ^19^, quienes afirman que el tratamiento idóneo para el odontoma compuesto es su eliminación lo más pronto posible, a fin de evitar posibles complicaciones posteriores ^13^^,^^19^, entre las cuales está la presencia de un diente impactado ^7^^,^^8^.

La paciente presenta una impactación del incisivo central superior derecho permanente (1.1), el cual es una condición poco frecuente; la impactación de un incisivo maxilar permanente oscila entre el 0,06% y el 1% de la población ^4^^-^^6^. Se decide tratar a la paciente inmediatamente, ya que, como se mencionó anteriormente e indican Vainer ^2^ y Kumar et al. ^5^, las impactaciones de incisivos permanentes pueden ocasionar problemas secundarios ^2^^,^^5^. Se coincide con estudios anteriores que afirman que el tratamiento ideal en caso de un incisivo impactado es la exposición quirúrgica y su tracción ortodóncica, posterior a la toma de una tomografía axial computarizada de la zona ^1^^,^^5^^,^^7^^,^^8^.

La tracción de un incisivo superior impactado es un reto para cualquier ortodoncista, ya que, además de colocar el diente en su posición, debe preocuparse por el componente estético del tejido duro y blando que lo rodea. En la mayoría de los casos clínicos estudiados ^1^^,^^2^^,^^4^^,^^5^^,^^8^^,^^10^^,^^12^^-^^17^ se dan opciones de tratamiento con aparatología fija de *brackets* ortodóncicos; sin embargo, a la hora de iniciar el tratamiento, esta paciente tiene 6 años y 4 meses de edad, presenta únicamente 4 dientes permanentes en proceso de erupción (1.6, 2.1, 2.2 y 2.6) y las demás piezas dentales son deciduas, lo que descarta el utilizar ortodoncia fija con *brackets* para traccionar el diente impactado. Por lo tanto, se decidió utilizar un aparato removible tipo Hawley modificado.

Por último, no se concuerda con Chaushu *et al*. ^7^ ni Chang *et al.*^10^, quienes afirman que la tracción de un incisivo maxilar permanente es un tratamiento de mucho tiempo, hasta de 2 años ^7^^,^^10^. En esta paciente, la tracción ortodóncica del 1.1 (incisivo central maxilar derecho permanente) se obtuvo en un tiempo total de 8 meses. El éxito y la disminución en el tiempo esperado se relacionan con el diagnóstico temprano y la corta edad de la paciente.

Este artículo no cuenta con fuentes de financiamiento institucionales ni presenta posibles conflictos de interés en cuanto a relación o condición que pudiera afectar su interpretación objetiva (institucional o económica).

## Fuente de financiamiento:

Autofinanciado

## References

[1] Marcolin P, Silveira E, Santos K (2018). Tracionamento de incisivo central superior impactado com aparelho removível relato de dois casos clínicos. Rev. Fac. Odontol Porto Alegre.

[2] Vainer D, Castro C (2010). Reporte de casos: retención de piezas dentales por odontomas. iDental.

[3] Grover PS, Lorton L (1985). The incidence of unerupted permanent teeth and related clinical cases. Oral Surg Oral Med Oral Pathol.

[4] Pavoni C, Franchi L, Laganà G, Baccetti T, Cozza P (2013). Management of impacted incisors following surgery to remove obstacles to eruption a prospective clinical trial. Pediatr Dent.

[5] Kumar P, Krishnam R, Srinivas K, Venkataramana V, Yugandhar G (2012). Traction of horizontally impacted central incisor a case report. Clinical and Surgical Techniques. Annals and Essences of Dentistry.

[6] Zaib J, Amin T, Gupta A, Singh R, Jamwal A (2013). Impacted maxillary incisors causes, diagnosis and management. Journal of Dental and Medical Sciences.

[7] Chaushu S, Becker T, Becker A (2015). Impacted central incisors factors affecting prognosis and treatment duration. Am J Orthod Dentofacial Orthop.

[8] Chandhoke TK, Agarwal S, Feldman J, Shah RA, Upadhyay M, Nanda R (2014). An efficient biomechanical approach for the management of an impacted maxillary central incisor. Am J Orthod Dentofacial Orthop.

[9] Tan C, Ekambaram M, Yiu CKY (2018). Prevalence, characteristic features, and complications associated with the occurrence of unerupted permanent incisors. PLoS One.

[10] Chang NY, Park JH, Kim SC, Kang KH, Cho JH, Cho JW, Jang HE, Chae JM (2016). Forced eruption of impacted maxillary central incisors with severely dilacerated roots. Am J Orthod Dentofacial Orthop.

[11] Shi X, Xie X, Quan J, Wang X, Sun X, Zhang C, Zheng S (2015). Evaluation of root and alveolar bone development of unilateral osseous impacted immature maxillary central incisors after the closed-eruption technique. Am J Orthod Dentofacial Orthop.

[12] Bastos VAS, Freitas-Fernandes LB, Soares DN, Neto OC, Abrahao AC, Farinhas JA, Fidalgo TK, Guimaraes LS (2018). Management of over retention of permanent incisor impacted by compound odontoma Clinical, radiological, and microscopic evaluation. Pediatric Dental Journal.

[13] Niharika P, Saha S, Soni KS, Saha S, Sarkar S (2019). Orthosurgical management of impacted maxillary central incisor associated with compound odontome: a case report. Int J Dent Health Sci.

[14] Estrada A, Katagiri M (2017). Orthodontic-surgical treatment of an impacted central incisor Case report. Rev Mex Ortod.

[15] Thosar NR, Vibhute P (2006). Surgical and orthodontic treatment of an impacted permanent central incisor a case report. J Indian Soc Pedod Prev Dent.

[16] Khan N, Shrivastava N, Shrivastava TV, Samadi FM (2014). An unusual case of compound odontome associated with maxillary impacted central incisor. Natl J Maxillofac Surg.

[17] Dhiman S, Khan S, Maheshwari S, Verma SK, Tariq M (2015). Orthosurgical management of odontome-associated maxillary central incisor impaction. J Dent Health Oral Disord Ther.

[18] Palacios DE, Guzma´n B, Miranda JE, Ramos CA (2016). Odontoma compuesto: revisión de la literatura y reporte de un caso con 40 dentículos. Revista ADM.

[19] Brenes JL (2013). Odontoma compuesto diagnóstico radiográfico y manejo quirúrgico. Reporte de dos casos clínicos. Rev. Cient Odontol.

[20] Martinovic G, Santorcuato B, Alister JP, Plaza C, Raffo J (2017). Odontoma compuesto diagnóstico y tratamiento. Reporte de casos & revisión de la literatura. Int J. Odontostomat.

